# The Effect of Naoxintong Capsule in the Treatment of Patients with Cerebral Infarction and Carotid Atherosclerosis: A Systematic Review and Meta-Analysis of Randomized Trials

**DOI:** 10.1155/2018/5892306

**Published:** 2018-07-24

**Authors:** Qiuer Liang, Yunfei Cai, Ruixue Chen, Weihao Chen, Liguo Chen, Ya Xiao

**Affiliations:** ^1^School of Traditional Chinese Medicine, Jinan University, Guangzhou, China; ^2^The Second Affiliated Hospital, Guangzhou Medical University, Guangzhou, China

## Abstract

**Objective:**

Naoxintong capsule (NXT) has been widely used to treat patients with cerebral infarction and carotid atherosclerosis. However, it is uncertain whether there is robust evidence on the effects of NXT for cerebral infarction and carotid atherosclerosis. A systematic review and meta-analysis of randomized trials were performed to assess the efficacy of NXT in the treatment of cerebral infarction and carotid atherosclerosis.

**Methods:**

The Cochrane Library, EMBASE, the Medline database, the Wanfang database, the China National Knowledge Infrastructure, and the VIP database were searched up to January 2018 with no language restrictions. Study selection, data extraction, quality assessment, and data analyses were performed according to the Cochrane standards.

**Results:**

Eleven studies (N=1141) in total satisfied the inclusion criteria for the meta-analysis. The results of meta-analysis showed that compared with the conventional therapy alone, NXT combined with conventional therapy could significantly improve national institutes of health stroke scale (NIHSS) score (MD= -3.92, 95%CI: -4.31~-3.52,* P*<0.00001), plaque area (MD= -0.16, 95%CI: -0.20~-0.13,* P*<0.00001), carotid intima-media thickness (IMT) (MD= -0.23, 95%CI: -0.26~-0.20,* P*<0.00001), total cholesterol (TC) (MD= -0.16, 95%CI: -0.79~-0.42,* P*<0.00001), triglyceride (TG) (MD= -0.69, 95%CI: -0.88~-0.51,* P*<0.00001), high-density lipoprotein cholesterol (HDL-C) (MD= 0.23, 95%CI: 0.15~0.31,* P*<0.00001), and low-density lipoprotein cholesterol (LDL-C) (MD= -0.42, 95%CI: -0.58~-0.25,* P*<0.00001). There were no reported adverse events in the studies.

**Conclusions:**

NXT is an effective and safe therapy option for patients with cerebral infarction and carotid atherosclerosis. However, due to the high clinical heterogeneity and small sample size of the included trials, further standardized preparation, large-scale and rigorously designed trials are needed.

## 1. Introduction

Cerebral infarction (CI), accounting for 60-80% of stroke, is the most common cerebrovascular disorder worldwide and also is a major cause of fatality and disability [1–4]. The pathogenesis of CI is complex and the main risk factors of CI are diabetes mellitus, hypertension, hyperlipidemia, atrial fibrillation, carotid atherosclerosis, alcohol consumption, cigarette smoking, and so forth [5]. In addition, a previous research suggested that 71.8% of ischemic cerebrovascular patients had carotid atherosclerotic plaque and increased intimal medial thickness [6]. Therefore, paying more attention to patients with CI and carotid atherosclerosis is of great significance in the prevention and treatment of CI.

Currently, the conventional therapy for CI and carotid atherosclerosis includes thrombolysis, antiplatelet aggregation, cerebral protection agents, preventing and treating complications, and controlling hypertension and hyperlipidemia [7, 8]. However, in spite of some beneficial effects, the rates of mortality and disability especially in patients with CI and carotid atherosclerosis still remain high. Therefore, development of new agents for patients with CI and carotid atherosclerosis are desirable.

Traditional Chinese medicine (TCM) has been used to treat stroke in China for more than 2,000 years. Advanced pharmaceutical technologies have led to the development of many oral agents of traditional Chinese patent medicines based on famous traditional formulae for the prevention and treatment of stroke. Naoxintong Capsule (NXT) is derived from a classic traditional Chinese prescription named Bu Yang Huan Wu decoction, which is widely used to treat patients with stroke in China. In recent years, more and more clinical studies indicated that NXT can not only ameliorate brain function and promote the recovery of consciousness, but also reduce the plaque area and carotid intima-media thickness [9, 10]. However, there is no comprehensive and systematic evidence to confirm its clinical efficacy in patients with CI and carotid atherosclerosis. Therefore, we conducted a comprehensive systematic review and meta-analysis of randomized trials to evaluate the effect of NXT for the treatment of patients with CI and carotid atherosclerosis.

## 2. Methods

### 2.1. Search Strategy

A comprehensive literature search was performed using Cochrane Library (1993 to January 2018), the China National Knowledge Infrastructure database (1979 to January 2018), the Wanfang database (1982 to January 2018), the VIP database (1989 to January 2018), and the Medline database (1989 to January 2018). The search terms used were (CI OR cerebral infarction) AND (carotid OR carotid artery disease OR carotid artery disorders OR carotid artery disorder OR carotid artery OR carotid atherosclerosis OR carotid atherosclerotic disease) AND Naoxintong. No limit was placed on language. Manual searches of conference compilations supplemented electronic searches.

### 2.2. Study Selection

Studies were considered to be eligible for inclusion if they met all of the following criteria. (i) The study was performed as a randomized controlled trial (RCT). (ii) Patients included in the study were diagnosed with cerebral infarction and carotid atherosclerosis. (iii) The study compared NXT plus conventional therapy with conventional therapy alone. (iv) Outcomes included at least one of the following: national institutes of health stroke scale (NIHSS), plaque area, intima-media thickness (IMT), total cholesterol (TC), triglyceride (TG), low-density lipoprotein cholesterol (LDL), and high-density lipoprotein cholesterol (HDL).

### 2.3. Data Extraction

Two researchers independently extracted data, including study design, randomization, blinding and subject characteristics, and duration of treatment. Disagreements were resolved after discussion with other investigators.

### 2.4. Data Analysis

Meta-analysis was carried out using Review Manager (RevMan 5.3), provided by the Cochrane Collaboration. Continuous data were presented as mean difference (MD), with 95% confidence interval (*CI*). The chi-squared test for heterogeneity was performed, and heterogeneity was presented as significant when* I*^*2*^ is over 50% or* P*< 0.1. Random effect model was used for the meta-analysis if there was significant heterogeneity, and fixed effect model was used when the heterogeneity was not significant.

## 3. Results

### 3.1. Search Results

**Figure 1:**
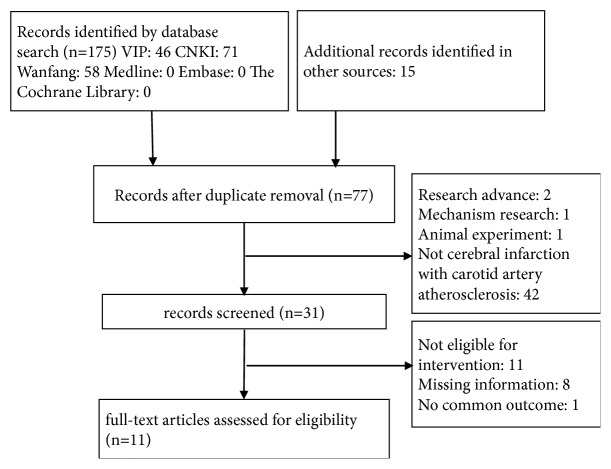
Flow chart of study selection process.

A total of 190 relevant studies were identified by computer search. Of these, 113 articles were duplicates and 46 articles were excluded on review of abstracts. After further reviewing, 11 studies (N=1141) satisfied the inclusion criteria for the meta-analysis [11–21].  Figure 1 is a flow chart of the study selection process. The general characteristics of included studies were clarified in Table 1.

### 3.2. The Quality Assessment of Included Studies

**Figure 2:**
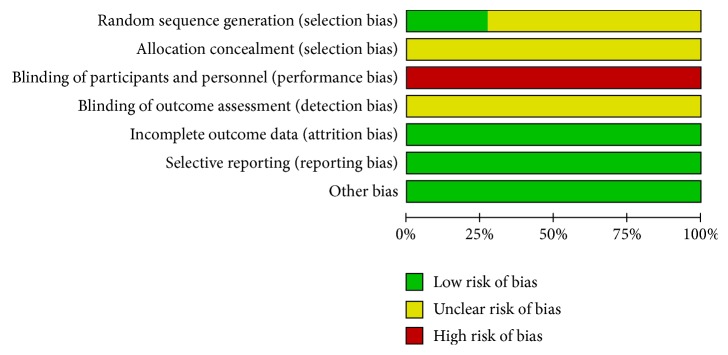
Risk of bias assessment.

The risk of bias assessment in the trials was summarized in Figure 2. In the aspect of random sequence generation, only 3 studies [11, 16, 21] used random number table, while 8 studies mentioned “random” but without details of randomization method. None of the 11 studies mentioned the allocation concealment. No studies mentioned the blinding of participants and personnel as well as blinding of outcome assessment. Low risk of bias was found across studies for incomplete outcome data and selective outcome reporting.

### 3.3. The Effects of Interventions

#### 3.3.1. NIHSS Score

**Figure 3:**
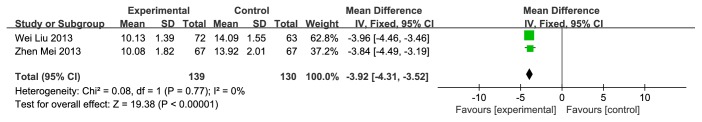
Forest plot of the effect of NXT on NIHSS score.

Two trials with 269 cases reported NIHSS score [18, 19]. NXT combined with conventional therapy showed significant improvement in NIHSS score compared with conventional therapy alone (MD= -3.92, 95%CI: -4.31~-3.52,* P*<0.00001) (Figure 3). No substantial heterogeneity was found (*P*=0.77,* I*^2^=0%).

#### 3.3.2. Plaque Area

**Figure 4:**
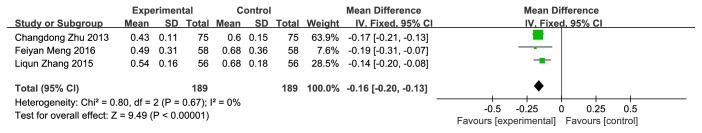
Forest plot of the effect of NXT on plaque area.

In the 11 included trials, 3 trials [15, 17, 21] with 378 cases reported plaque area. Compared with conventional therapy alone, NXT combined with conventional therapy showed significant improvement in plaque area (MD= -0.16, 95%CI: -0.20~-0.13, P<0.00001), with no significant heterogeneity between the studies (*P*=0.67,* I*^2^=0%) (Figure 4).

#### 3.3.3. IMT

**Figure 5:**
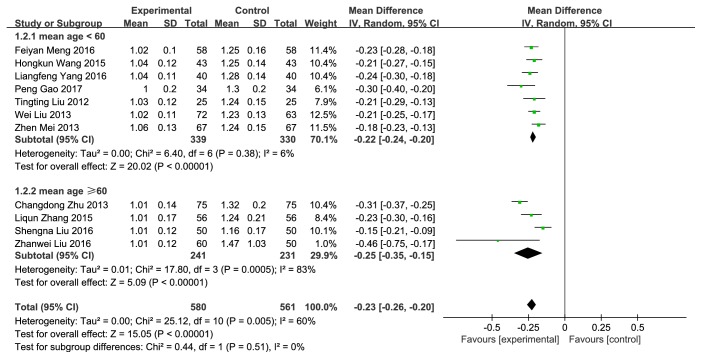
Forest plot of the effect of NXT on IMT.

**Figure 6:**
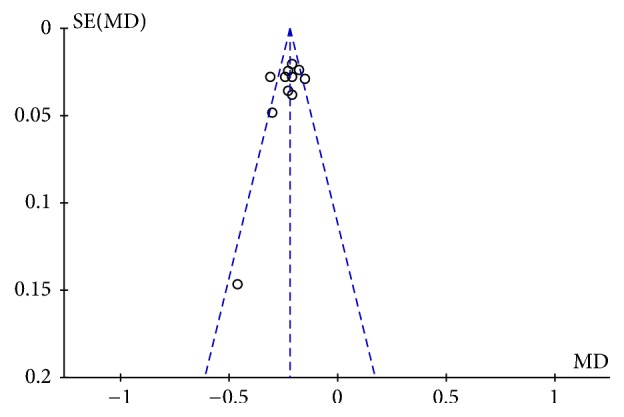
The funnel plot of the effect of NXT on IMT.

10 trails with 1141 cases reported IMT [11–21]. Compared with conventional therapy alone, NXT combined with conventional therapy showed significant improvement in IMT (MD= -0.23, 95%CI: -0.26~-0.20,* P*<0.00001), with significant heterogeneity between the studies (*P*=0.005,* I*^*2*^=60%) (Figure 5). Due to the high statistical heterogeneity, we carried out a subgroup analysis based on mean age. The results showed that when the mean age was less than 60, the heterogeneity was significantly reduced (*P*=0.38,* I*^*2*^=6%), indicating that the mean age may be an important source of the heterogeneity. We also used funnel plot to evaluate the publication bias. The results showed that the distribution is nearly symmetric in general (Figure 6).

#### 3.3.4. TC

**Figure 7:**
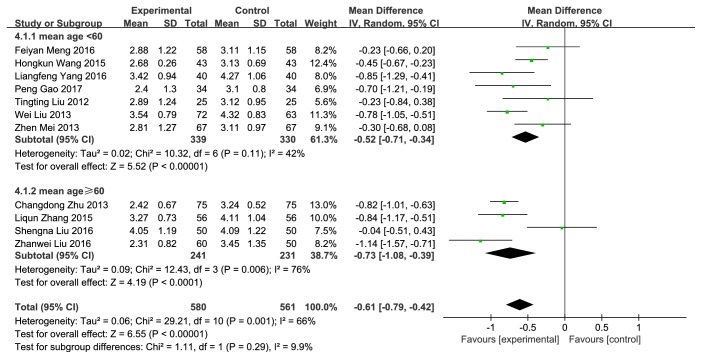
Forest plot of the effect of NXT on TC.

11 trials with 1141 cases reported TC [11–21]. NXT combined with conventional therapy was significantly more likely to reduce the level of TC (MD = −0.61, 95%CI: −0.79~−0.42,* P*<0.00001) than conventional therapy alone (Figure 7). The heterogeneity was significant (*P* = 0.001,* I*^2^ = 66%). We also performed a subgroup analysis based on mean age. The heterogeneity did not have much change after subgroup analysis.

#### 3.3.5. TG

**Figure 8:**
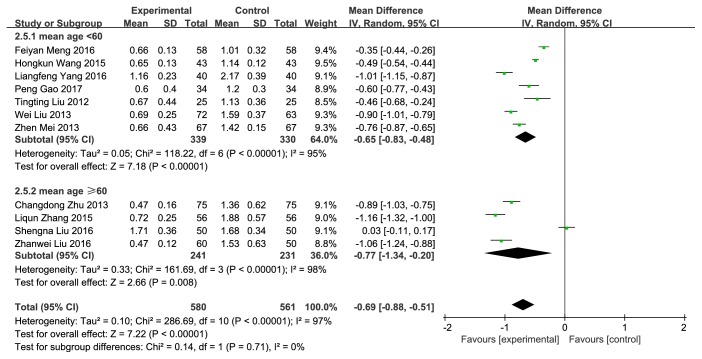
Forest plot of the effect of NXT on TG.

11 trials with 1141 cases reported TG [11–21]. NXT combined with conventional therapy was significantly more likely to reduce the level of TG (MD = −0.69, 95%CI: −0.88~−0.51,* P*<0.00001) than conventional therapy alone (Figure 8). The heterogeneity was significant (*P*<0.00001,* I*^*2*^=97%). We conducted a subgroup analysis based on mean age. Although the subgroup analysis was performed, the high heterogeneity remained.

#### 3.3.6. HDL

**Figure 9:**
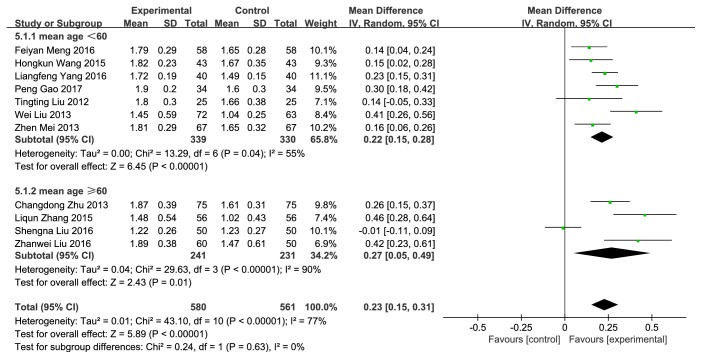
Forest plot of the effect of NXT on HDL.

11 trials with 1141 cases reported HDL [11–21]. Compared with conventional therapy alone, NXT combined with conventional therapy showed significant improvement in HDL (MD= 0.23, 95%CI:0.15~0.31,* P*<0.00001), with significant heterogeneity between the studies (*P*<0.00001,* I*^*2*^=77%) (Figure 9). We performed a subgroup analysis based on mean age. The heterogeneity did not have much change after subgroup analysis.

#### 3.3.7. LDL

**Figure 10:**
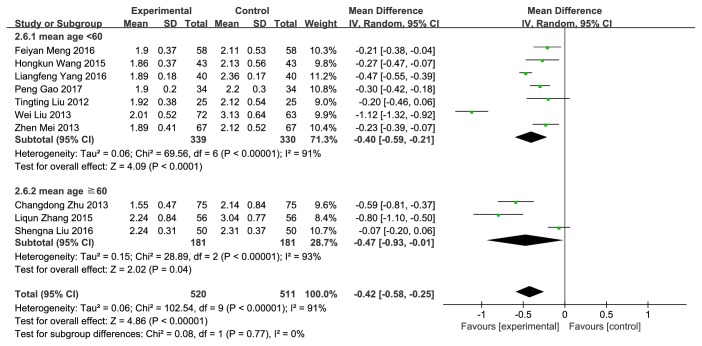
Forest plot of the effect of NXT on LDL.

10 trials with 1031 cases reported LDL [11, 12, 14–21]. NXT combined with conventional therapy was significantly more likely to reduce the level of LDL (MD= -0.42, 95%CI: -0.58~-0.25,* P*<0.00001) than conventional therapy alone (Figure 10). The heterogeneity was significant (*P*<0.00001,* I*^*2*^=91%). We conducted a subgroup analysis based on mean age. Although the subgroup analysis was performed, the high heterogeneity remained.

## 4. Discussion

The outcomes of the current meta-analysis mainly included NIHSS score, plaque area, IMT, and lipid level. NIHSS is a scale for clinical evaluation of neurological deficits in patients with acute cerebral infarction and has 15 items. The score of NIHSS ranges from 0 to 42 [22]. When the score was over 12, the incidence of proximal occlusion of the middle cerebral artery and cerebral infarction increased [23]. NIHSS can not only assess different aspects of neural function, but also evaluate the symptoms and signs of nervous system [24]. The results of our meta-analysis revealed that NXT combined with conventional therapy is more efficacious than conventional therapy alone in improving NIHSS score. Plaque area and IMT are commonly used as indicators of carotid atherosclerosis and the unstable plaque associated with high lipid levels can lead to intracranial vascular blockage which can result in stroke [25, 26]. The results of our meta-analysis suggested that NXT combined with conventional therapy showed significant improvement not only in plaque area and IMT but also in lipid levels. Furthermore, there were no reported adverse events in the studies. It seems that NXT is an effective and safe therapy option for patients with cerebral infarction and carotid atherosclerosis.

In TCM, “Qi deficiency and blood stasis” is considered to be the important pathogenic factor for patients with CI and carotid atherosclerosis [27, 28]. NXT is composed of sixteen herbs which has the function of replenishing qi and promoting blood circulation (Astragalus membranaceus, Radix paeoniae rubra, Salvia miltiorrhiza, Ligusticum wallichii, Angelica sinensis, Peach kernel, Carthami Flos, Olibanum, Myrrh, etc.). Experimental data has demonstrated that NXT had protective effect against cerebral ischemia reperfusion injury associated with the downregulation of LOX-1, pERK1/2, and NF-*κ*B expression [29]. NXT could improve neurological deficits and reduce cerebral infarct size glutamine by regulating monoamine neurotransmitter metabolism, amino acid metabolism, energy metabolism, and lipid metabolism [30]. In addition, NXT can inhibit the advanced atherosclerosis and enhance the plaque stability in apolipoprotein E deficient mice [31]. The effectiveness of NXT in the treatment of CI and carotid atherosclerosis might be due to different functions of the single plant extracts. A previous study has shown that calycosin isolated from the roots of Astragalus membranaceus can ameliorate neurologic deficit and infarct volume by reducing the content of malondialdehyde (MDA), protein carbonyl, and reactive oxygen species (ROS) and upregulating the activities of superoxide dismutase (SOD), catalase, and glutathione peroxidase (GSH-Px) [32]. Paeoniflorin has been demonstrated to have neuroprotective effect on cerebral ischemic rat by activating adenosine A1 receptor [33]. Salvianolic acid B extracted from Salvia miltiorrhiza showed a protective action against the ischemia reperfusion induced injury in rat brain by reducing lipid peroxides [34]. Z-Ligustilide can ameliorate brain damage induced by permanent forebrain ischemia through an antioxidant effect and improved cholinergic activity [35].

However, this meta-analysis had several limitations. First, the quality of included studies was generally not high. No trial was identified as a multicenter, large sample, prospective, double-blinded, controlled randomized trial. Although all included studies had a randomization design, only three trials [11, 16, 21] described the method of randomization procedure. None of the included studies mentioned the allocation concealment and no studies mentioned the blinding of participants and personnel as well as blinding of outcome assessment, which might create the potential selection bias and performance bias. It needed to be demonstrated whether the effect of NXT would remain the same when applied in future large-scale, double-blinded, controlled randomized trials. Second, all the patients in the included studies were treated for 6 months under controlled conditions. The treatment duration was not long enough to evaluate the long term clinical effect of NXT. Third, the heterogeneity was significant for most of the outcomes. We performed a subgroup analysis based on the mean age and found that the age of the patients included in the studies may be one of the sources of the heterogeneity. Fourth, since all of the included studies were published in Chinese, the possibility of publication bias existed.

In summary, the systematic review and meta-analysis suggested that NXT combined with conventional therapy is more efficacious than conventional therapy alone in the treatment of patients with CI and carotid atherosclerosis. However, due to the high clinical heterogeneity and small sample size of the included trials, large-scale, randomized double-blind, multicenter trials are required.

## Figures and Tables

**Table 1:** The characteristics of the included studies.

Author(s), year	Sample size	Mean age (year)		Interventions		Duration (month)
		Control	Experimental	Control	Experimental	
Liu 2016 [11]	100	69±2.01	68±2.39	Atorvastatin 20mg/d Aspirin 100mg/d	Atorvastatin 20mg/d Aspirin 100mg/d Naoxintong (1.6g, TID)	6
Gao 2017 [12]	68	56.7±8.2	56.7±8.2	Atorvastatin 20mg/d Aspirin 100mg/d	Atorvastatin 20mg/d Aspirin 100mg/d Naoxintong (1.6g,TID)	6
Liu 2016 [13]	110	62.32±5.36	62.32±5.36	Atorvastatin 20mg/d Aspirin 100mg/d	Atorvastatin 20mg/d Aspirin 100mg/d Naoxintong (1.6g,TID)	6
Liu 2012 [14]	50	59.2±16.8	59.2±16.8	Atorvastatin 20mg/d Aspirin 100mg/d	Atorvastatin 20mg/d Aspirin 100mg/d Naoxintong (1.6g,TID)	6
Meng 2016 [15]	116	56±8.5	55±9.5	Atorvastatin 20mg/d Aspirin 100mg/d	Atorvastatin 20mg/d Aspirin 100mg/d Naoxintong (1.6g,TID)	6
Wang 2015 [16]	86	57.3±2.5	57.3±2.5	Atorvastatin 20mg/d Aspirin 100mg/d	Atorvastatin 20mg/d Aspirin 100mg/d Naoxintong (1.6g,TID)	6
Zhang 2015 [17]	112	63.3±4.2	63.7±5.8	Atorvastatin 20mg/d Aspirin 100mg/d	Atorvastatin 20mg/d Aspirin 100mg/d Naoxintong (1.6g,TID)	6
Liu 2013 [18]	135	55.1±8.2	55.9±8.4	Atorvastatin 20mg/d Aspirin 100mg/d	Atorvastatin 20mg/d Aspirin 100mg/d Naoxintong (1.6g,TID)	6
Mei 2013 [19]	134	56.3±5.8	58.7±7.1	Atorvastatin 20mg/d Aspirin 100mg/d	Atorvastatin 20mg/d Aspirin 100mg/d Naoxintong (1.6g,TID)	6
Yang 2016 [20]	80	56±15.6	54.2±14.6	Atorvastatin 20mg/d Aspirin 100mg/d	Atorvastatin 20mg/d Aspirin 100mg/d Naoxintong (1.6g,TID)	6
Zhu 2013 [21]	150	62.39±5.69	62.39±5.69	Atorvastatin 20mg/d Aspirin 100mg/d	Atorvastatin 20mg/d Aspirin 100mg/d Naoxintong (1.6g,TID)	6

## Data Availability

The data supporting this systematic review and meta-analysis are from previously reported studies and datasets, which have been cited.
